# Responses of β-thalassemia and compound heterozygote of Sickle/βthalassemia of BCL11A Gene Polymorphism in Pakistani Patients

**DOI:** 10.12669/pjms.39.6.7183

**Published:** 2023

**Authors:** Nayab Soomro, Mohsin Wahid, Mehreen Mehmood, Syed Hasan Danish

**Affiliations:** 1 Nayab Soomro, Dow Research Institute of Biotechnology and Biomedical Sciences, Dow University of Health Sciences Karachi, Karachi, Pakistan; 2 Mohsin Wahid, Dow Research Institute of Biotechnology and Biomedical Sciences, Department of Pathology, Dow International Medical College, Dow University of Health Sciences Karachi, Pakistan; 3 Mehreen Mehmood, Department of Pathology, Dow International Medical College, Dow University of Health Sciences Karachi, Karachi, Pakistan; 4 Syed Hasan Danish, Department of Community Medicine, Ziauddin University, Karachi, Pakistan

**Keywords:** Beta thalassemia, Compound heterozygote of sickle-beta thalassemia. Single Nucleotide Polymorphisms, Sanger Sequencing

## Abstract

**Background and Objective:**

Beta-thalassemia major (β-Thal) and compound heterozygote of Sickle β-thalassemia (S-β Thal) are hereditary autosomal recessive disorders resulting from mutations or deletion in β-globin gene cluster. Patients with increased HbF levels having polymorphism at BCL11A site loci have shown clinical significance. The present study aimed to assess the frequency of BCL11A gene polymorphism in a study population of β-Thal, S-β Thal & Controls using Sanger sequencing leading to plot the HbF response of polymorphism with reference to wild type.

**Methods:**

The sample size of the study is n=180, groups were divided in Controls, β-thal & S-β thal. One ml blood was drawn from patients and controls to extract DNA for PCR amplification and BCL11A locus genotyping using Sanger sequencing. This study was carried out at Dow Research Institute of Biotechnology and Biomedical Sciences, for one year from March 2021 to February 2022.

**Results:**

The HbF response of three groups is hyperbolic with 83 for β-Thal, 16 for S-β Thal and close to zero for controls. The frequency of heterozygous variant GA of BCL11A gene polymorphism is 51%. The frequency of homozygous variant GG is 49%. Complete absence of wild type AA in patient group. The frequency of BCL11A polymorphism in control group was 43% (with male 18% and female 21%) showing wild type status of 57%.

**Conclusions:**

The patient groups of SCD and Beta thalassemia are devoid of wild type status. The wild type status of BCL11A is 57% even in control population. Higher level of HbF in B-thalassemia and SCD and B Thalassemia is a cost-effective screening marker before switching to an expensive genotyping testing.

## INTRODUCTION

Hemoglobinopathies are classified as single gene disorders; however, they show significant clinical heterogeneity and phenotypic variability. Therefore, investigating molecular genetics in the heritable sub-types of these disorders, for each population specifically, would be helpful for personalized therapeutics.^1^ The most common type of genetic variation in the human genome is caused by single nucleotide substitutions which produces single nucleotide variants. If two or more alternative DNA variants of this type are present in more than 1% of the population, it is described as single nucleotide polymorphism (SNP). Genetic polymorphisms usually do not directly cause the disease but they may work as leading factor towards disease severity.^2^

Fetal hemoglobin (HbF) is considered as an important genetic modifier of β-thalassemia phenotype and its levels that continue to exist in adulthood has shown to have significant effect on disease severity in sickle cell disease and the beta-thalassemia syndromes.^3^ Several polymorphisms located in genetic loci that are linked with HbF regulation have been identified and screened in different populations.^1^ The levels of fetal hemoglobin (HbF) have shown to be affected by SNPs at Xmn1, BCL11A and HSB1L-MYB genes.^4^ The association between elevated HbF levels with upstream of β-globin gene cluster are identified in several populations.^3^ One variation in the intergenic region of the BCL11A gene is directly implicated in HbF gene silence in adulthood.^5^ Studies done on genetic association have shown that sequence variations in BCL11A gene are linked with altered HbF levels^6^ BCL11A is considered as a potent regulator of HbF production and its reduced levels have shown to delay the switching of fetal to adult hemoglobin.^7^

The present study aimed to assess the frequency of BCL11A gene polymorphism in a study population of β-Thal, S-β Thal & Controls using Sanger sequencing leading to plot the HbF response of polymorphism with reference to wild type.

This study was designed for identification of rs4671393 (BCL11A) gene polymorphism in beta-thalassemia major and compound heterozygotes of HbS/β-thalassemia in Pakistani patients and establishing an association between HbF levels and the identified SNP. There is limited data available on studies involving Single Nucleotide Polymorphisms in thalassemia patients in Pakistan. Also, most work is done on PCR based methods and not including Sequencing technique which is considered as a gold standard. There is no study in the country to the best of our knowledge, which has investigated the gene polymorphism in BCL.11A (rs4671393) in both Beta-thalassemia and Beta Thalassemia -SCD (compound heterozygotes) Pakistani patients and used Sanger sequencing for the anlaysis.

## METHODS

The study was approved by Institutions Review Board (IRB-1434/DUHS/Approval/2019) on 19/12/19, and informed consent was obtained from all candidates included in the study. The study was carried out at Dow Research Institute of Biotechnology and Biomedical Sciences (DRIBBS), Dow University of Health Sciences, Karachi, Pakistan from March 2021 to February 2022.

All patients were grouped according to their hematological parameters including, HbF levels, analyzed by HPLC. The sample size of the study was n=180, divided in three groups of 60 each. One ml of peripheral blood samples from the three groups, A (Control 60 samples), B (Beta thalassemia major 60 samples), C (compound heterozygote of sickle beta thalassemia 60 samples) were used to extract genomic DNA. Extracted DNA was used for PCR, gel electrophoresis and PCR purification. PCR reaction mixture was made in 25µl final volume. PCR conditions were denaturation at 94°C for 30 second and annealing at 60°C for one minute and extension at 72°C for one minute. Thermal cycler was used for PCR (Bio-Rad, USA). For gel electrophoresis, 2% agarose gel was prepared. The amplicon size of each amplified PCR product corresponded to its size on DNA ladder. The amplicon size of rs4671393 was approximately (415) bp.

The purified PCR products were then sent for Sanger sequencing commercially. The sequenced data were aligned with Qiagen CLC Genomics workbench software available at Dow Research Institute of Biotechnology and Biomedical Sciences and their association with HbF levels was analyzed using SPSS version 24.

## RESULTS

We had a total of n=180 samples, out of which 151 were selected for sequencing based on the quality of the extracted DNA and successful DNA amplification on PCR. The results showed that out of 151, n=104 samples were found to have the BCL11A polymorphism. The distribution included n=17(34%) in β-thalassemia group, Compound heterozygote of Sickle –β- thalassemia group n=42 (40.3%), and Control group n=45 (43.26%). In Control group n=18(40%) were males and n=27(60%) were females. Sickle-β- thalassemia group had n=24(57.14%) males and n=18(42.85%) females, while the third group β-thalassemia major had n=8(47%) males and n=9 (53%) females. Results of polymorphism in rs4671393 in intron two of the BCL11A gene in the B-thalassemia and Sickle -Beta groups using Sanger sequencing demonstrated that n=51 (49%) were homozygous (G/G) and n= 53(51%) were heterozygous (G/A).

Descriptive statistics showed that HbF levels were 83.52±16.49, for Sickle-β- thalassemia group HbF was 16.03±10.84 and for control group HbF was 0.34±.19. Among β-thalassemia group there were n=12 (70.6%) homozygous and n=5 (29.4%) heterozygous participants. Sickle-β-thalassemia group comprised of n=30 (71.4%) homozygous and n= 12 (28.6%) heterozygous however in the control group there were n= 9 (20%) homozygous and n=26 (80%) heterozygous. When association was seen by chi square between Gene polymorphisms and groups it was found statistically significant (p value 0.000)

Since our data was non parametric so Kruskal Wallis test was applied and it was observed that the differences between HbF levels among all three groups were statistically significant, (P.value 0.000). When seen among genotypes in the Homozygous group differences among the three groups results were statistically significant (p value 0.000). Similarly, in the heterozygous group the differences between the three groups were statistically significant (p value 0.000).

In our study 1 SNP of BCL11A gene was analyzed (rs4671393) using Sanger sequencing. Information associated with this SNV has been listed below in Table-I. Alleles and genotypes percentage of rs4671393 in BCL11A gene have been displayed in Table-II.

**Table-I:** Sanger sequencing associated with SNV in BCL11A gene

Groups	SNP ID	Location	Position	Allele (Forward Orientation)
Control			168,169	A>G A>G
β-thal	Rs4671393	Intron 2	167,169,170	A>G A>G
S-β			168,169	A>G G>A

**Table-II:** Alleles and genotypes percentage of rs4671393 in BCL11A gene.

Gene(chromosome) BCL11A(2p16.1	Single-nucleotide polymorphism locus	Genotype Frequency	Allele Frequency
Control group	rs4671393	GG-10 G/A-35	G-55 A-35
Beta Thalassemia major	rs4671393	GG-12 G/A-5	G-29 A-5
Compound Heterozygote Sickle-β-thalassemia	rs4671393	GG-30 G/A-12	G-72 A-12

Sanger sequencing of polymorphism rs4671393 of this gene revealed the lack of variation C>T.

**Table-III:** Comparison of HbF levels between genotypes.

BCL11A gene	Groups	N	Mean rank	P
Homozygous	β-thalassemia major	12	45.50	0.000
	S-β- thalassemia	30	24.50	
	Control	9	5.00	
Heterozygous	β-thalassemia major	5	51.00	0.000
	S-β- thalassemia	12	42.42	
	Control	36	18.53	

**Table-IV:** Distribution of genotype of BCL11A gene polymorphism and of HbF levels in Control, B-thalassemia, S-B-thalassemia.

Genotype	Specification	GG	GA	Total	P
β-thalassemia major	No. of Patients	12 (70.59%)	5 (29.41%)	17	0.000
	HbF levels (g/dl) (mean ± SD)	8.262±25.867	0.909±0.014		
S-β- thalassemia	No. of Patients	30 (71.43%)	12 (28.57%)	42	0.000
	HbF levels (g/dl) (mean ± SD)	16.66±11.52	15.02±0.105		
Control	No. of Patients	10 (22.22%)	35 (77.78%)	45	0.000

In summary, the important observations from this study are:

1. The HbF response of three groups is hyperbolic with 83 for β-Thal, 16 for S-β Thal and close to zero for controls.
2. The frequency of heterozygous variant GA of BCL11A gene polymorphism is 51%.
3. The frequency of homozygous variant GG is 49%.
4. Complete absence of wild type AA in patient group.
5. The frequency of BCL11A polymorphism in control group is 43% (with male 18% and female 21%) showing a wild type status of 57%.

## DISCUSSION

Fetal hemoglobin (HbF) is considered as a potent genetic modifier of β-Thalassemia and Sickle cell disease phenotype.^8^ Patients with high HbF levels have shown to have longer life expectancy and lesser complications.^9^ It has been shown that three genetic loci that carry DNA polymorphisms in BCL11A, β-globin and *HBS1L-MYB* genes are involved in modulation of HbF levels.^9^ Several studies have shown the association between BCL11A single nucleotide polymorphisms and diseases severity in β- Thalassemia major and sickle cell disease.^10^ The main goal of this study combined detection of SNP variants by Sanger sequencing at rs4671393 and their association with increased HbF levels with the intension to re-evaluate blood transfusion requirements, drug administration and potential modification in the treatment plan. In this regard, multiple researches have been done in different countries but limited data is available in Pakistan on the polymorphisms and their association with HbF levels.

One of the studies conducted in Pakistan, investigated modulatory effects of SNPs in Xmn1, BCL11A and *HBS1L-MYB* genes in β-Thalassemia major and intermedia patients and found an association.^11^ The specific BCL11A polymorphism that they investigated was (rs766432).

In our study, we have performed Sanger sequencing on compound heterozygotes of Sickle beta thalassemia and Beta thalassemia patients to determine the different variants of target polymorphism (rs4671393) in BCL11A gene. There is no data available on this specific polymorphism in compound heterozygote of Sickle beta thalassemia patient in Pakistan. Based upon the polymorphisms identified by Sanger Sequencing, polymorphism-specific therapies could be taken into consideration for Sickle beta thalassemia as well as beta thalassemia major patients.

In another study, specific SNPs in Xmn1 and BCL11A genes were correlated with Hydroxyurea (HU) response in Pakistani β-Thalassemia patients and found that although BCL11A polymorphisms were more prevalent in the Hydroxyurea (HU) responders, their association with HU response was not found to be statistically significant.^12^ Other studies investigated Xmn1 polymorphisms in Pakistani thalassemia patients using polymerase chain reaction.^13^-^16^

The presence of rs4671393 Polymorphisms in mentioned patients (Control, B-thalassemia, S-B-thalassemia), has been compared with their fetal hemoglobin levels by Kruskal-Wallis test. We found that there is clear association between the presences of allele G which found in majority of cases, has effect on increase in HbF levels. Allele A which also has correlation with HbF in our both groups B-thalassemia & S-β-thalassemia. We discovered that the homozygous variation G/G was less prevalent in our cohort, occurring at a frequency of n=51(49%). The heterozygous variation G/A was more prevalent, accounting for n=53 (51%) of all successfully sequenced cases.

### Limitations:

The samples which either yielded low amount of DNA or had failed PCR and had no bands on Gel electrophoresis could have been replaced with additional samples but could not be done due to budget and timeline restrictions.

## CONCLUSION

We concluded that there is association of these alleles with increased HbF levels in β-thalassemia major and Compound heterozygotes of Sickle-β- thalassemia diseases in Pakistani cohort. The patient groups of SCD and Beta thalassemia are devoid of wild type status. The wild type status of BCL11A is 57% even in control population. Higher level of HbF in B-thalassemia and SCD and β-Thalassemia is a cost-effective screening marker before switching to an expensive genotyping testing.

### Authors’ Contribution:

**MW** designed and supervised the study including all the experiments, anlaysis and final review of the manuscript.

**NS** collected the clinical data and performed all laboratory procedures under supervision and prepared the paper.

**MM** provided HPLC diagnosed patients sample, history and examination and reviewed the paper.

**SHD** did the data analysis and provided statistical support and reviewed the manuscript. All authors have approved the final version of the manuscript.

**MW** is the corresponding author and responsible and accountable for the accuracy or integrity of the work.
